# Ten Simple Rules for Effective Statistical Practice

**DOI:** 10.1371/journal.pcbi.1004961

**Published:** 2016-06-09

**Authors:** Robert E. Kass, Brian S. Caffo, Marie Davidian, Xiao-Li Meng, Bin Yu, Nancy Reid

**Affiliations:** 1 Department of Statistics, Machine Learning Department, and Center for the Neural Basis of Cognition, Carnegie Mellon University, Pittsburgh, Pennsylvania, United States of America; 2 Department of Biostatistics, Bloomberg School of Public Health, Johns Hopkins University, Baltimore, Maryland, United States of America; 3 Department of Statistics, North Carolina State University, Raleigh, North Carolina, United States of America; 4 Department of Statistics, Harvard University, Cambridge, Massachusetts, United States of America; 5 Department of Statistics and Department of Electrical Engineering and Computer Science, University of California Berkeley, Berkeley, California, United States of America; 6 Department of Statistical Sciences, University of Toronto, Toronto, Ontario, Canada; Whitehead Institute, UNITED STATES

## Introduction

Several months ago, Phil Bourne, the initiator and frequent author of the wildly successful and incredibly useful “Ten Simple Rules” series, suggested that some statisticians put together a Ten Simple Rules article related to statistics. (One of the rules for writing a PLOS Ten Simple Rules article is to be Phil Bourne [1]. In lieu of that, we hope effusive praise for Phil will suffice.)

Implicit in the guidelines for writing Ten Simple Rules [1] is “know your audience.” We developed our list of rules with researchers in mind: researchers having some knowledge of statistics, possibly with one or more statisticians available in their building, or possibly with a healthy do-it-yourself attitude and a handful of statistical packages on their laptops. We drew on our experience in both collaborative research and teaching, and, it must be said, from our frustration at being asked, more than once, to “take a quick look at my student’s thesis/my grant application/my referee’s report: it needs some input on the stats, but it should be pretty straightforward.”

There are some outstanding resources available that explain many of these concepts clearly and in much more detail than we have been able to do here: among our favorites are Cox and Donnelly [2], Leek [3], Peng [4], Kass et al. [5], Tukey [6], and Yu [7].

Every article on statistics requires at least one caveat. Here is ours: we refer in this article to “science” as a convenient shorthand for investigations using data to study questions of interest. This includes social science, engineering, digital humanities, finance, and so on. Statisticians are not shy about reminding administrators that statistical science has an impact on nearly every part of almost all organizations.

## Rule 1: Statistical Methods Should Enable Data to Answer Scientific Questions

A big difference between inexperienced users of statistics and expert statisticians appears as soon as they contemplate the uses of some data. While it is obvious that experiments generate data to answer scientific questions, inexperienced users of statistics tend to take for granted the link between data and scientific issues and, as a result, may jump directly to a technique based on data structure rather than scientific goal. For example, if the data were in a table, as for microarray gene expression data, they might look for a method by asking, “Which test should I use?” while a more experienced person would, instead, start with the underlying question, such as, “Where are the differentiated genes?” and, from there, would consider multiple ways the data might provide answers. Perhaps a formal statistical test would be useful, but other approaches might be applied as alternatives, such as heat maps or clustering techniques. Similarly, in neuroimaging, understanding brain activity under various experimental conditions is the main goal; illustrating this with nice images is secondary. This shift in perspective from statistical technique to scientific question may change the way one approaches data collection and analysis. After learning about the questions, statistical experts discuss with their scientific collaborators the ways that data might answer these questions and, thus, what kinds of studies might be most useful. Together, they try to identify potential sources of variability and what hidden realities could break the hypothesized links between data and scientific inferences; only then do they develop analytic goals and strategies. This is a major reason why collaborating with statisticians can be helpful, and also why the collaborative process works best when initiated early in an investigation. See Rule 3.

## Rule 2: Signals Always Come with Noise

Grappling with variability is central to the discipline of statistics. Variability comes in many forms. In some cases variability is good, because we need variability in predictors to explain variability in outcomes. For example, to determine if smoking is associated with lung cancer, we need variability in smoking habits; to find genetic associations with diseases, we need genetic variation. Other times variability may be annoying, such as when we get three different numbers when measuring the same thing three times. This latter variability is usually called “noise,” in the sense that it is either not understood or thought to be irrelevant. Statistical analyses aim to assess the signal provided by the data, the interesting variability, in the presence of noise, or irrelevant variability.

A starting point for many statistical procedures is to introduce a mathematical abstraction: outcomes, such as patients being diagnosed with specific diseases or receiving numerical scores on diagnostic tests, will vary across the set of individuals being studied, and statistical formalism describes such variation using probability distributions. Thus, for example, a data histogram might be replaced, in theory, by a probability distribution, thereby shifting attention from the raw data to the numerical parameters that determine the precise features of the probability distribution, such as its shape, its spread, or the location of its center. Probability distributions are used in statistical models, with the model specifying the way signal and noise get combined in producing the data we observe, or would like to observe. This fundamental step makes statistical inferences possible. Without it, every data value would be considered unique, and we would be left trying to figure out all the detailed processes that might cause an instrument to give different values when measuring the same thing several times. Conceptualizing signal and noise in terms of probability within statistical models has proven to be an extremely effective simplification, allowing us to capture the variability in data in order to express uncertainty about quantities we are trying to understand. The formalism can also help by directing us to look for likely sources of systematic error, known as bias.

Big data makes these issues more important, not less. For example, Google Flu Trends debuted to great excitement in 2008, but turned out to overestimate the prevalence of influenza by nearly 50%, largely due to bias caused by the way the data were collected; see Harford [8], for example.

## Rule 3: Plan Ahead, Really Ahead

When substantial effort will be involved in collecting data, statistical issues may not be captured in an isolated statistical question such as, “What should my *n* be?” As we suggested in Rule 1, rather than focusing on a specific detail in the design of the experiment, someone with a lot of statistical experience is likely to step back and consider many aspects of data collection in the context of overall goals and may start by asking, “What would be the ideal outcome of your experiment, and how would you interpret it?” In trying to determine whether observations of X and Y tend to vary together, as opposed to independently, key issues would involve the way X and Y are measured, the extent to which the measurements represent the underlying conceptual meanings of X and Y, the many factors that could affect the measurements, the ability to control those factors, and whether some of those factors might introduce systematic errors (bias).

In Rule 2 we pointed out that statistical models help link data to goals by shifting attention to theoretical quantities of interest. For example, in making electrophysiological measurements from a pair of neurons, a neurobiologist may take for granted a particular measurement methodology along with the supposition that these two neurons will represent a whole class of similar neurons under similar experimental conditions. On the other hand, a statistician will immediately wonder how the specific measurements get at the issue of co-variation; what the major influences on the measurements are, and whether some of them can be eliminated by clever experimental design; what causes variation among repeated measurements, and how quantitative knowledge about sources of variation might influence data collection; and whether these neurons may be considered to be sampled from a well-defined population, and how the process of picking that pair could influence subsequent statistical analyses. A conversation that covers such basic issues may reveal possibilities an experimenter has not yet considered.

Asking questions at the design stage can save headaches at the analysis stage: careful data collection can greatly simplify analysis and make it more rigorous. Or, as Sir Ronald Fisher put it: “To consult the statistician after an experiment is finished is often merely to ask him to conduct a post mortem examination. He can perhaps say what the experiment died of” [9]. As a good starting point for reading on planning of investigations, see Chapters 1 through 4 of [2].

## Rule 4: Worry about Data Quality

Well-trained experimenters understand instinctively that, when it comes to data analysis, “garbage in produces garbage out.” However, the complexity of modern data collection requires many assumptions about the function of technology, often including data pre-processing technology. It is highly advisable to approach pre-processing with care, as it can have profound effects that easily go unnoticed.

Even with pre-processed data, further considerable effort may be needed prior to analysis; this is variously called “data cleaning,” “data munging,” or “data carpentry.” Hands-on experience can be extremely useful, as data cleaning often reveals important concerns about data quality, in the best case confirming that what was measured is indeed what was intended to be measured and, in the worst case, ensuring that losses are cut early.

Units of measurement should be understood and recorded consistently. It is important that missing data values can be recognized as such by relevant software. For example, 999 may signify the number 999, or it could be code for “we have no clue.” There should be a defensible rule for handling situations such as “non-detects,” and data should be scanned for anomalies such as variable 27 having half its values equal to 0.00027. Try to understand as much as you can how these data arrived at your desk or disk. Why are some data missing or incomplete? Did they get lost through some substantively relevant mechanism? Understanding such mechanisms can help to avoid some seriously misleading results. For example, in a developmental imaging study of attention deficit hyperactivity disorder, might some data have been lost from children with the most severe hyperactivity because they could not sit still in the MR scanner?

Once the data have been wrestled into a convenient format, have a look! Tinkering around with the data, also known as exploratory data analysis, is often the most informative part of the analysis. Exploratory plots can reveal data quality issues and outliers. Simple summaries, such as means, standard deviations, and quantiles, can help refine thinking and offer face validity checks for hypotheses. Many studies, especially when going in completely new scientific directions, are exploratory by design; the area may be too novel to include clear a priori hypotheses. Working with the data informally can help generate new hypotheses and ideas. However, it is also important to acknowledge the specific ways data are selected prior to formal analyses and to consider how such selection might affect conclusions. And it is important to remember that using a single set of data to both generate and test hypotheses is problematic. See Rule 9.

## Rule 5: Statistical Analysis Is More Than a Set of Computations

Statistical software provides tools to assist analyses, not define them. The scientific context is critical, and the key to principled statistical analysis is to bring analytic methods into close correspondence with scientific questions. See Rule 1. While it can be helpful to include references to a specific algorithm or piece of software in the Methods section of a paper, this should not be a substitute for an explanation of the choice of statistical method in answering a question. A reader will likely want to consider the fundamental issue of whether the analytic technique is appropriately linked to the substantive questions being answered. Don’t make the reader puzzle over this: spell it out clearly.

At the same time, a structured algorithmic approach to the steps in your analysis can be very helpful in making this analysis reproducible by yourself at a later time, or by others with the same or similar data. See Rule 10.

## Rule 6: Keep it Simple

All else being equal, simplicity trumps complexity. This rule has been rediscovered and enshrined in operating procedures across many domains and variously described as “Occam’s razor,” “KISS,” “less is more,” and “simplicity is the ultimate sophistication.” The principle of parsimony can be a trusted guide: start with simple approaches and only add complexity as needed, and then only add as little as seems essential.

Having said this, scientific data have detailed structure, and simple models can’t always accommodate important intricacies. The common assumption of independence is often incorrect and nearly always needs careful examination. See Rule 8. Large numbers of measurements, interactions among explanatory variables, nonlinear mechanisms of action, missing data, confounding, sampling biases, and so on, can all require an increase in model complexity.

Keep in mind that good design, implemented well, can often allow simple methods of analysis to produce strong results. See Rule 3. Simple models help us to create order out of complex phenomena, and simple models are well suited for communication to our colleagues and the wider world.

## Rule 7: Provide Assessments of Variability

Nearly all biological measurements, when repeated, exhibit substantial variation, and this creates uncertainty in the result of every calculation based on the data. A basic purpose of statistical analysis is to help assess uncertainty, often in the form of a standard error or confidence interval, and one of the great successes of statistical modeling and inference is that it can provide estimates of standard errors from the same data that produce estimates of the quantity of interest. When reporting results, it is essential to supply some notion of statistical uncertainty. A common mistake is to calculate standard errors without taking into account the dependencies among data or variables, which usually means a substantial underestimate of the real uncertainty. See Rule 8.

Remember that every number obtained from the data by some computation would change somewhat, even if the measurements were repeated on the same biological material. If you are using new material, you can add to the measurement variability an increase due to the natural variability among samples. If you are collecting data on a different day, in a different lab, or under a slightly changed protocol, there are now three more potential sources of variability to be accounted for. In microarray analysis, batch effects are well known to introduce extra variability, and several methods are available to filter these. Extra variability means extra uncertainty in the conclusions, and this uncertainty needs to be reported. Such reporting is invaluable for planning the next investigation.

It is a very common feature of big data that uncertainty assessments tend to be overly optimistic (Cox [10], Meng [11]). For an instructive, and beguilingly simple, quantitative analysis most relevant to surveys, see the “data defect” section of [11]. Big data is not always as big as it looks: a large number of measurements on a small number of samples requires very careful estimation of the standard error, not least because these measurements are quite likely to be dependent.

## Rule 8: Check Your Assumptions

Every statistical inference involves assumptions, which are based on substantive knowledge and some probabilistic representation of data variation—this is what we call a statistical model. Even the so-called “model-free” techniques require assumptions, albeit less restrictive assumptions, so this terminology is somewhat misleading.

The most common statistical methods involve an assumption of linear relationships. For example, the ordinary correlation coefficient, also called the Pearson correlation, is a measure of linear association. Linearity often works well as a first approximation or as a depiction of a general trend, especially when the amount of noise in the data makes it difficult to distinguish between linear and nonlinear relationships. However, for any given set of data, the appropriateness of the linear model is an empirical issue and should be investigated.

In many ways, a more worrisome, and very common, assumption in statistical analysis is that multiple observations in the data are statistically independent. This is worrisome because relatively small deviations from this assumption can have drastic effects. When measurements are made across time, for example, the temporal sequencing may be important; if it is, specialized methods appropriate for time series need to be considered.

In addition to nonlinearity and statistical dependence, missing data, systematic biases in measurements, and a variety of other factors can cause violations of statistical modeling assumptions, even in the best experiments. Widely available statistical software makes it easy to perform analyses without careful attention to inherent assumptions, and this risks inaccurate, or even misleading, results. It is therefore important to understand the assumptions embodied in the methods you are using and to do whatever you can to understand and assess those assumptions. At a minimum, you will want to check how well your statistical model fits the data. Visual displays and plots of data and residuals from fitting are helpful for evaluating the relevance of assumptions and the fit of the model, and some basic techniques for assessing model fit are available in most statistical software. Remember, though, that several models can “pass the fit test” on the same data. See Rule 1 and Rule 6.

## Rule 9: When Possible, Replicate!

Every good analyst examines the data at great length, looking for patterns of many types and searching for predicted and unpredicted results. This process often involves dozens of procedures, including many alternative visualizations and a host of numerical slices through the data. Eventually, some particular features of the data are deemed interesting and important, and these are often the results reported in the resulting publication.

When statistical inferences, such as *p*-values, follow extensive looks at the data, they no longer have their usual interpretation. Ignoring this reality is dishonest: it is like painting a bull’s eye around the landing spot of your arrow. This is known in some circles as *p*-hacking, and much has been written about its perils and pitfalls: see, for example, [12] and [13].

Recently there has been a great deal of criticism of the use of *p*-values in science, largely related to the misperception that results can’t be worthy of publication unless “*p* is less than 0.05.” The recent statement from the American Statistical Association (ASA) [14] presents a detailed view of the merits and limitations of the *p*-value.

Statisticians tend to be aware of the most obvious kinds of data snooping, such as choosing particular variables for a reported analysis, and there are methods that can help adjust results in these cases; the False Discovery Rate method of Benjamini and Hochberg [15] is the basis for several of these.

For some analyses, there may be a case that some kinds of preliminary data manipulation are likely to be innocuous. In other situations, analysts may build into their work an informal check by trusting only extremely small *p*-values. For example, in high energy physics, the requirement of a “5-sigma” result is at least partly an approximate correction for what is called the “look-elsewhere effect.”

The only truly reliable solution to the problem posed by data snooping is to record the statistical inference procedures that produced the key results, together with the features of the data to which they were applied, and then to replicate the same analysis using new data. Independent replications of this type often go a step further by introducing modifications to the experimental protocol, so that the replication will also provide some degree of robustness to experimental details.

Ideally, replication is performed by an independent investigator. The scientific results that stand the test of time are those that get confirmed across a variety of different, but closely related, situations. In the absence of experimental replications, appropriate forms of data perturbation can be helpful (Yu [16]). In many contexts, complete replication is very difficult or impossible, as in large-scale experiments such as multi-center clinical trials. In such cases, a minimum standard would be to follow Rule 10.

## Rule 10: Make Your Analysis Reproducible

In our current framework for publication of scientific results, the independent replication discussed in Rule 9 is not practical for most investigators. A different standard, which is easier to achieve, is reproducibility: given the same set of data, together with a complete description of the analysis, it should be possible to reproduce the tables, figures, and statistical inferences. However, even this lower standard can face multiple barriers, such as different computing architectures, software versions, and settings.

One can dramatically improve the ability to reproduce findings by being very systematic about the steps in the analysis (see Rule 5), by sharing the data and code used to produce the results, and by following Goodman et al. [17]. Modern reproducible research tools like Sweave [18], knitr [19], and iPython [20] notebooks take this a step further and combine the research report with the code. Reproducible research is itself an ongoing area of research and a very important area that we all need to pay attention to.

## Conclusion

Mark Twain popularized the saying, “There are three kinds of lies: lies, damned lies, and statistics.” It is true that data are frequently used selectively to give arguments a false sense of support. Knowingly misusing data or concealing important information about the way data and data summaries have been obtained is, of course, highly unethical. More insidious, however, are the widespread instances of claims made about scientific hypotheses based on well-intentioned yet faulty statistical reasoning. One of our chief aims here has been to emphasize succinctly many of the origins of such problems and ways to avoid the pitfalls.

A central and common task for us as research investigators is to decipher what our data are able to say about the problems we are trying to solve. Statistics is a language constructed to assist this process, with probability as its grammar. While rudimentary conversations are possible without good command of the language (and are conducted routinely), principled statistical analysis is critical in grappling with many subtle phenomena to ensure that nothing serious will be lost in translation and to increase the likelihood that your research findings will stand the test of time. To achieve full fluency in this mathematically sophisticated language requires years of training and practice, but we hope the Ten Simple Rules laid out here will provide some essential guidelines.

Among the many articles reporting on the ASA’s statement on *p-*values, we particularly liked a quote from biostatistician Andrew Vickers in [21]: “Treat statistics as a science, not a recipe.” This is a great candidate for Rule 0.
